# Ileocolic Intussusception in an Adult Due to Inflammatory Fibroid Polyp: A Case Report

**DOI:** 10.7759/cureus.31098

**Published:** 2022-11-04

**Authors:** Archana Khanduri, Shreshtha Singh, Harshdeep Tyagi, Parikshit Morey, Rahul Gupta

**Affiliations:** 1 Gastrointestinal Surgery, Synergy Institute of Medical Sciences, Dehradun, IND; 2 Anaesthesiology, Synergy Institute of Medical Sciences, Dehradun, IND; 3 Radiology, Synergy Institute of Medical Sciences, Dehradun, IND

**Keywords:** small-bowel obstruction, right hemicolectomy, ileocolonic intussusception, vanek's tumor, inflammatory fibroid polyp

## Abstract

The ileocolic region is the most common site of intussusception. It occurs mostly in children but can also occur in adults. Ileal tumors and polyps can cause ileocolic intussusception. Inflammatory fibroid polyp (IFP) is one of the rarest benign tumors of the gastrointestinal tract. The most common sites of IFP are the stomach, ileum, colon, duodenum, and esophagus. Small IFPs can be managed endoscopically but larger IFPs and those with complications such as intussusception require surgical resection. We report a case of ileal IFP presenting with small bowel obstruction due to ileocolic intussusception. The patient was successfully treated by surgical excision. The diagnosis of IFP was made based on a histopathological examination of the resected specimen.

## Introduction

Intussusception is common in children but rare in adults [1,2]. The main cause of intussusception in children is idiopathic and responds to conservative treatment in most cases. The conservative treatment includes water-soluble contrast enema, air enema, or ultrasound-guided hydrostatic reduction [3]. However, more than two-thirds of adult intussusceptions are due to underlying bowel pathology such as tumor, Meckel’s diverticulum, and polyps [2,4]. It often causes acute or chronic intermittent bowel obstruction [3]. Preoperative diagnosis is often missed or delayed due to non-specific symptoms [1]. Abdominal imaging can help in making a timely diagnosis of intussusception but the lead point may not be visible [5]. Most of the adult patients require surgical intervention.

An inflammatory fibroid polyp (IFP) is a rare benign tumor of the gastrointestinal tract. It is most frequently located in the stomach and responsible for 0.1% of all gastric polyps [6]. Small intestinal IFPs are extremely rare with only case reports and series reported in the literature [4,7]. Most of the reported cases of IFP have been sporadic. However, there are few reports of familial IFPs [8,9]. We report a case of ileal IFP causing ileocolic intussusception leading to small bowel obstruction and treated successfully by surgery.

## Case presentation

**Figure 1:**
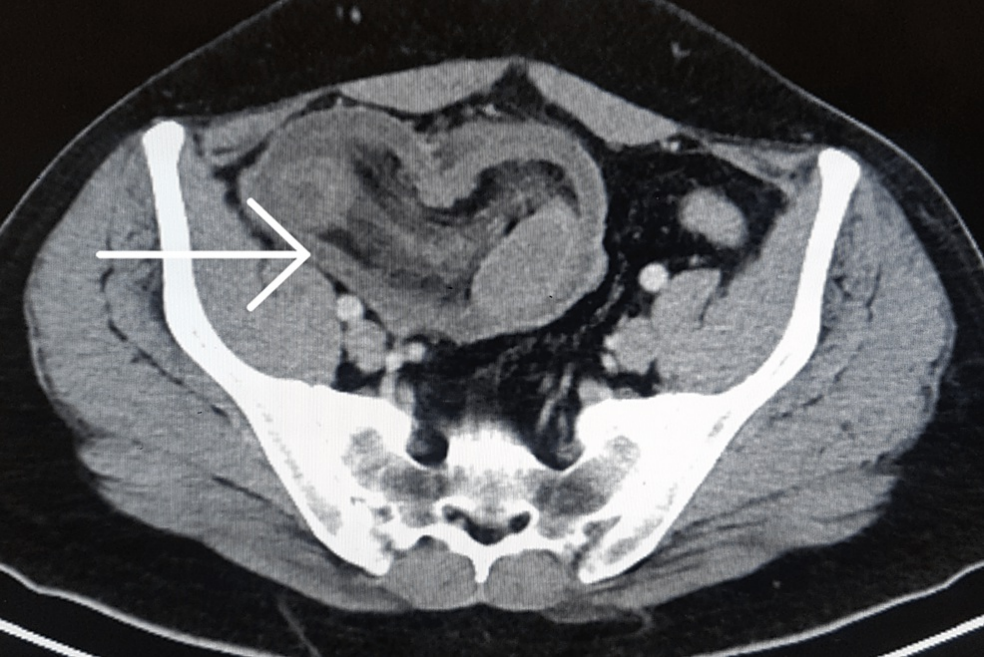
Contrast-enhanced CT of the abdomen and pelvis showing the ileocolic intussusception.

A 41-year-old male with a known case of type II diabetes mellitus presented to our emergency department with sudden onset of abdominal pain, nausea, vomiting, and obstipation for one day. His medical and family history was non-contributory. Physical examination revealed an ill-defined, tender lump in the right lumbar region. Routine blood investigations including complete hemogram, and liver and renal function tests were within normal limits. The differential diagnoses included intestinal obstruction due to appendicular or cecal mass. Hence, contrast-enhanced CT (CECT) of the abdomen was performed. CECT abdomen revealed an oval mass-like structure with a bowel within bowel concentric ring-like appearance in the ileocolic region suggestive of intussusception (Figure 1). 

**Figure 2:**
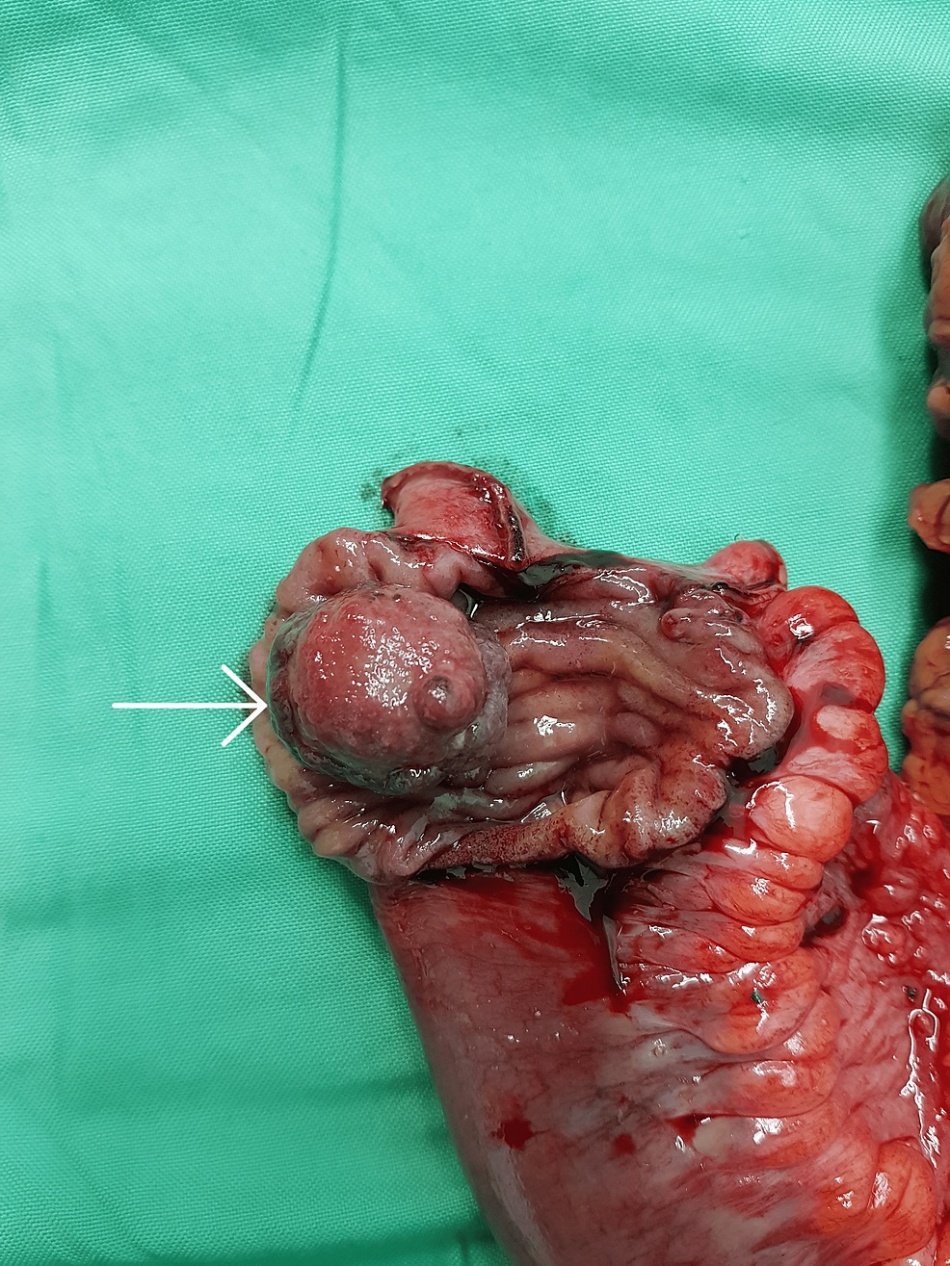
Gross appearance of the ileal inflammatory fibroid polyp.

Additionally, there was proximal small bowel dilatation and mild ascites. The patient was kept nil orally, a nasogastric tube was inserted for gastrointestinal decompression, and intravenous fluids were given. In view of intestinal obstruction with ileocolic intussusception, surgery was planned. The patient underwent diagnostic laparoscopy with laparoscopic-assisted open right hemicolectomy with ileocolonic anastomosis. At diagnostic laparoscopy, a 3 x 3 cm polypoidal lesion was found in the terminal ileum which was acting as the lead point of the intussusception (Figure 2). 

**Figure 3:**
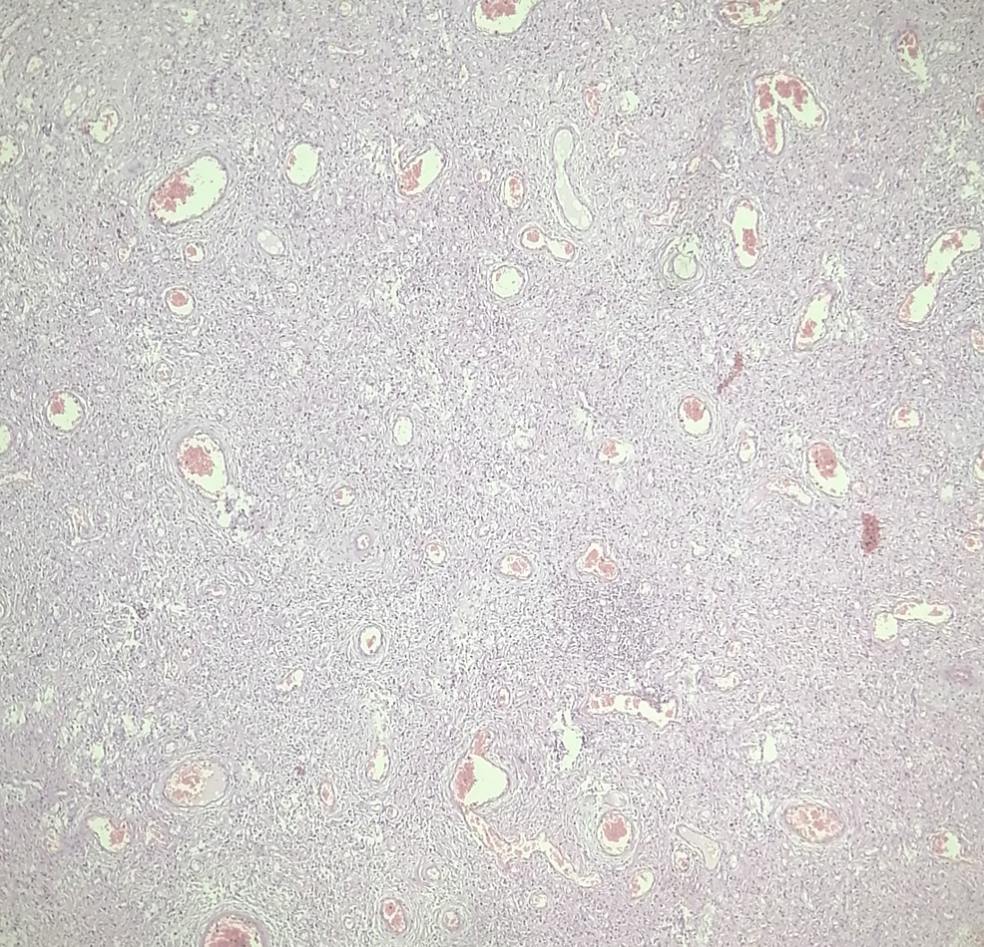
Microscopic appearance of the ileal polyp showing the fibrovascular stroma with inflammatory infiltrates suggestive of inflammatory fibroid polyp (H & E, 20x).

Multiple enlarged mesenteric lymph nodes were present in the ileocecal region which were excised en-bloc during radical right hemicolectomy. After resection, side-to-side ileocolonic hand-sewn anastomosis was performed. The operative time was 160 minutes and the estimated blood loss was 100 ml. There were no intraoperative complications. The postoperative recovery was uneventful with a hospital stay of seven days. Histopathological analysis of the polypoidal lesion showed focally attenuated mucosal lining with underlying spindle stroma, fibrovascular areas with inflammatory infiltrates, and hemosiderin-laden macrophages (Figure 3). There was no evidence of cellular atypia, pleomorphism, or granuloma. The appendix showed luminal fecolith and lymphoid hyperplasia. All fourteen resected lymph nodes showed reactive changes and were negative for metastasis. The resected intestinal margins were unremarkable. Based on these findings, the final diagnosis of IFP was made. The patient was followed up five months after surgery for abdominal pain. No significant abnormality was seen on CECT abdomen and the patient responded to medical therapy. On telephonic contact at one year, the patient was symptom-free.

## Discussion

IFP is a benign tumor of the digestive tract first described by Vanek in 1949 as an eosinophilic submucosal granuloma [10]. The etiology of IFP is unknown. Triggering factors including foreign body, chronic pyloric infection, and parasitic infection have been suggested, but the evidence is still lacking. In view of marked eosinophilic infiltration in most cases, a localized variant of gastroenteritis has also been proposed as a possible etiology [11,12]. However, in the present case, there were non-specific inflammatory infiltrates. Poorly controlled inflammatory response to chemical, traumatic, and metabolic mucosal injury has also been hypothesized [12].

The most common site of IFP is the gastric antrum (70%) followed by the small bowel (20%) [1,7,12]. The rare sites of IFP are the rectum, duodenum, and esophagus (the distal third being the commonest site) [12,13]. The most common age of presentation is the fourth to the seventh decade of life as seen in the present case [7,12]. Clinical presentation depends on the size, location, and complications of IFP. Most of the gastric and colonic IFPs are incidentally detected during endoscopy or colonoscopy. Small intestinal IFPs most frequently present with intussusception causing abdominal pain as seen in the current case [7]. Macroscopically, IFPs may be polypoidal or sessile with an average size of around 4 cm. However giant IFPs up to 20 cm in diameter have also been reported [14]. 

Epigastric pain and bleeding are the most common symptoms of gastric IFP while colicky abdominal pain with constipation and abdominal distension are the most common symptoms of intestinal IFP [4,7,15]. In the index case, the patient presented with the clinical features of intestinal obstruction due to intussusception. 

About 70% to 90% of adult intussusceptions are due to benign or malignant neoplasms such as adenocarcinoma, lymphoma, IFP, lipoma, and adenoma. Small bowel lesions are not usually diagnosed pre-operatively because they present with vague symptoms of bowel obstruction due to intussusception. Laboratory investigations and abdominal radiographs are not helpful in making the diagnosis as they will demonstrate findings only in the presence of bowel obstruction [2,16].

Radiological diagnosis of intussusception is often made by ultrasound especially in the pediatric population [1,3]. In adults, the diagnosis is made by CECT or MRI [2,4,5]. These techniques have some limitations in determining the underlying pathology causing the intussusception but they provide excellent preoperative evaluation, including the possible extension and/or dissemination of a malignant tumor, and may also be useful in suggesting the presence of vascular compromise [5]. In the current case, the lead point of intussusception could not be appreciated on CECT. Small IFPs often go undetected on radiology. But large IFPs can be easily detected on cross-sectional imaging but often resemble other gastrointestinal tumors such as gastrointestinal stromal tumors, neuroendocrine tumors, lymphoma, and adenocarcinoma [14,16,17]. Hence, preoperative diagnosis of small bowel IFP is difficult as seen in the present case. 

The diagnosis of IFP is most often established on histological and immunohistochemical examination of the endoscopic or surgical specimen. Usually, IFPs stain positive for CD34 and vimentin. Occasionally, IFPs may also stain positive for smooth muscle actin, calponin, CD35, and cyclin D1 [7,18]. Characteristically, IFPs do not stain for CD117 and S100, which differentiates them from more common mesenchymal tumors such as gastrointestinal stromal tumors and nerve sheath tumors. IFPs are also not easily confirmed by biopsy sampling alone and up to 90% of biopsies are non-confirmatory [19]. The final diagnosis is only possible on histological analysis after complete surgical or endoscopic resection. 

As IFP is a benign disease, patients with small IFP diagnosed with endoscopic biopsy can be left alone if asymptomatic. However, most IFPs are symptomatic or there is suspicion of malignancy requiring surgical excision. Most of the previous studies have reported excellent outcomes with no recurrence on follow-up [7,15]. 

Ileal IFP causing intussusception is rare and very few cases have been reported in literature [16,17,19]. Among the reported cases, IFPs less than 5 cm were missed while those larger than 5 cm were suspected to be gastrointestinal stromal tumors on preoperative CECT [16,17]. All these patients underwent surgical excision similar to the present case and the diagnosis of IFP was confirmed on histopathology.

## Conclusions

IFP in the ileocecal region is rare and can lead to intussusception causing acute intestinal obstruction. It should be included in differential diagnosis while treating adult patients with intussusception or bowel obstruction. IFP mimics other gastrointestinal tumors such as stromal tumors, neuroendocrine tumors, lymphoma, and adenocarcinoma on preoperative radiological imaging. The final diagnosis can be made only on histopathology.
